# The potential and limitations of intrahepatic cholangiocyte organoids to study inborn errors of metabolism

**DOI:** 10.1002/jimd.12450

**Published:** 2021-11-03

**Authors:** Vivian Lehmann, Imre F. Schene, Arif I. Ardisasmita, Nalan Liv, Tineke Veenendaal, Judith Klumperman, Hubert P. J. van der Doef, Henkjan J. Verkade, Monique M. A. Verstegen, Luc J. W. van der Laan, Judith J. M. Jans, Nanda M. Verhoeven‐Duif, Peter M. van Hasselt, Edward E. S. Nieuwenhuis, Bart Spee, Sabine A. Fuchs

**Affiliations:** ^1^ Department of Metabolic Diseases University Medical Center Utrecht Utrecht The Netherlands; ^2^ Department of Veterinary Medicine Utrecht University Utrecht The Netherlands; ^3^ Section Cell Biology, Center for Molecular Medicine University Medical Center Utrecht Utrecht The Netherlands; ^4^ Department of Pediatric Gastroenterology University Medical Center Groningen Groningen The Netherlands; ^5^ Department of Hepatology University Medical Center Groningen Groningen The Netherlands; ^6^ Department of Surgery Erasmus MC‐University Medical Center Rotterdam The Netherlands; ^7^ Department of Metabolic Diagnostics University Medical Center Utrecht Utrecht The Netherlands

**Keywords:** cystic fibrosis, inborn errors of metabolism, intrahepatic cholangiocyte organoids, methylmalonic acidemia, patient‐specific *in vitro* modeling, Wilson disease

## Abstract

Inborn errors of metabolism (IEMs) comprise a diverse group of individually rare monogenic disorders that affect metabolic pathways. Mutations lead to enzymatic deficiency or dysfunction, which results in intermediate metabolite accumulation or deficit leading to disease phenotypes. Currently, treatment options for many IEMs are insufficient. Rarity of individual IEMs hampers therapy development and phenotypic and genetic heterogeneity suggest beneficial effects of personalized approaches. Recently, cultures of patient‐own liver‐derived intrahepatic cholangiocyte organoids (ICOs) have been established. Since most metabolic genes are expressed in the liver, patient‐derived ICOs represent exciting possibilities for *in vitro* modeling and personalized drug testing for IEMs. However, the exact application range of ICOs remains unclear. To address this, we examined which metabolic pathways can be studied with ICOs and what the potential and limitations of patient‐derived ICOs are to model metabolic functions. We present functional assays in patient ICOs with defects in branched‐chain amino acid metabolism (methylmalonic acidemia), copper metabolism (Wilson disease), and transporter defects (cystic fibrosis). We discuss the broad range of functional assays that can be applied to ICOs, but also address the limitations of these patient‐specific cell models. In doing so, we aim to guide the selection of the appropriate cell model for studies of a specific disease or metabolic process.

## 1 INTRODUCTION

Inborn errors of metabolism (IEMs) comprise a broad category of monogenic disorders affecting metabolic pathways, with a cumulative prevalence of approximately 1 in 2000 live births annually.^1^, ^2^ While current technological progress has increasingly improved early IEM diagnosis, full mechanistic understanding, and treatments beyond symptomatic approaches remain limited.^3^ To study IEMs and address the urgent need for novel treatments,^4^ human *in vitro* models, which recapitulate the patient's genetic make‐up and tissue function are needed.

In recent years, organoids have been increasingly employed to model various organs.^5^, ^6^, ^7^ Organoids are three‐dimensional (3D) cell cultures that can be established from adult stem cells, induced pluripotent stem cells or embryonic stem cells. Upon differentiation, organoids recapitulate cellular and functional aspects of the organ of origin and have been proposed for use as preclinical tools for personalized medicine.^8^, ^9^, ^10^, ^11^, ^12^

In 2015, liver organoids were generated from patient‐derived LGR5‐positive adult liver stem cells.^5^ To differentiate these organoids from other liver‐derived organoids, they have been named intrahepatic cholangiocyte‐derived liver organoids (ICOs).^6^, ^13^ ICOs recapitulate individual patients' genetic make‐up, retain tissue specific functions without the need for genetic reprograming, and can be differentiated toward hepatocyte‐like cells. Moreover, ICOs are suitable for long‐term culture, while remaining genetically stable.^6^

Patient derived ICOs have been used to phenotype human disease, including cancer, Alagille syndrome, and alpha‐1 antitrypsin deficiency.^6^, ^14^, ^15^, ^16^ However, it has also become clear that not all liver functions are well reflected in ICOs (Ardisasmita et al., submitted). Thus, the question remains which metabolic categories and IEMs can be studied with ICOs and whether ICOs represent advantages over other patient‐derived cell models such as fibroblasts.

To address this, we investigated the potential and limitations of patient‐derived ICOs to model metabolic functions. We describe the ease of establishing and expanding ICOs from small biopsies and present a range of functional assays that can be applied to ICOs. By also addressing current limitations in *in vitro* modeling, we hope to guide the selection process of the appropriate model to study IEMs.

## 2 MATERIALS AND METHODS

### 2.1 Organoid generation and culture

Liver tissue was obtained from explant tissue (0.5‐1 cm^3^) or needle biopsies (~5 mm^3^) after patient informed consent. Use for our studies was ethically approved by the different collaborating University Centers (MEC‐2014‐060; STEM 1‐402/K). Liver cells were isolated as described previously (Supporting Information).^6^ The pelleted digest was plated in 10 μL droplets of 70% (v/v) Matrigel (Corning) and cultured in seeding medium (SM) (Supporting Information). All cultures were kept in a humified atmosphere of 95% air and 5% CO_2_ at 37°C and media were refreshed every other day. Once ICOs formed, Noggin, Wnt conditioned media (CM), Y27632, and human embryonic stem cell cloning recovery solution (hES) were removed from the SM, now termed expansion medium (EM). ICO cultures were passaged 1:4 to 1:10 every 7 to 10 days by mechanical dissociation. For differentiation, ICOs were pretreated with 25 ng/mL BMP7 (Peprotech) for 3 days, whereafter media were changed to differentiation media (DM, Supporting Information). After 8 days of differentiation, ICOs were used for functional or gene expression analyses.

### 2.2 Immunohistochemistry

Wholemount immunohistochemistry on ICOs was performed as described (Supporting Information),^17^ using primary and secondary antibodies (Tables S1 and S2). Nuclei were stained with 0.5 μg/mL DAPI (Sigma‐Aldrich). Imaging was performed on a Leica TCS SP8 confocal microscope.

### 2.3 LC‐MS/MS

ICOs and culture media of one MMA patient and one HC were harvested and stored at −80°C until further use. Methanol was added to the culture medium to extract the acylcarnitines and free carnitine. For intracellular analyses, ICOs were bullet blended in ice cold methanol. Stable isotope internal standards (D3‐carnitine, D3‐C4‐carnitine, D3‐C8‐carnitine, and D3‐C16 carnitine) and acetonitrile were added and samples were vortexed and centrifuged (5 minutes, 12 000 rpm). The methanol eluate was evaporated under heated (40°C) nitrogen to dryness and butylated for 15 minutes at 60°C. Excess reagent was evaporated to dryness and residue was reconstituted in 100 μL acetonitrile. Concentrations of free carnitine and acylcarnitines were analyzed by flow injection using liquid chromatography (Alliance 2790, Waters) coupled to a Micromass QuattroUltima mass spectrometer (HPLC/MS/MS).^18^

### 2.4 Copper toxicity assay

ICOs of one HC and one Wilson disease patient were incubated with EM containing copper(II)chloride (CuCl_2_) for 72 hours. Cell viability was assessed by quantifying necrosis marker propidium iodide (0.1 mg/mL, Thermo‐Fisher) and DNA marker Hoechst (5 μg/mL, Sigma‐Aldrich) signals after 15 minutes of incubation at 37°C. For this, whole Matrigel droplets were imaged with an inverted Olympus IX53 epifluorescence microscope at 2× magnification and quantified with Fiji.

### 2.5 Forskolin‐induced swelling assay

ICOs of one cystic fibrosis transmembrane conductance regulator (CFTR) patient and two HCs were treated as described previously.^16^ Briefly, 1000 cells were seeded in 5 μL Matrigel droplets per well of a 96 wells plate and cultured for 3 days in EM with 10 μM Y27632. Thereafter, Y27632 and forskolin (FSK) were removed and culture proceeded for 3 days. Next, ICOs were treated with calcein green (10 μM, Invitrogen) for 30 minutes at 37°C, 0 to 10 μM FSK was added and ICOs were analyzed by confocal live‐cell microscopy (LSM710, Zeiss, 5× objective). For drug screening, patient ICOs were preincubated for 72 hours with 15 μM of VX‐809 (Selleck Chemicals LLC), while 15 μM of VX‐770 (Selleck Chemicals LLC) was added just before analysis. For CFTR inhibition a combination of 50 μM CFTR_inh_‐172 (Sigma) and 50 μM GlyH‐101 (Calbiochem) was added to respective conditions 3 hours before analysis. Foskolin‐induced swelling (FIS) of ICOs was automatically determined by quantifying total ICO area relative to *t* = 0 with Volocity imaging software (Improvision). After correcting for the average area of 0 μM FSK, each condition was analyzed in triplicate to determine the average area under the curve (AUC) using Graphpad Prism.

### 2.6 RNA sequencing and analysis

mRNA was isolated from two HC ICO cultures as described (Supporting Information). Raw sequencing data of primary healthy fibroblasts (GSM1306659, GSM3146360, GSM3067785, and GSM3067799) and healthy liver tissue (SAMN07109073, GSM3442821, and GSM3442822) were obtained from the European Nucleotide Archive (ENA). Next, data were processed and corrected for batch effects (Supporting Information). Heatmaps were generated using pheatmap package (v. 1.0.12) in R (v3.6.3). The log2 fold change relative to the mean expression of the gene across all fibroblast and ICO DM samples was determined. Finally, genes were sorted for best expression in DM ICOs and into metabolic categories based on categorizations of Vademecum Metabolicum, KEGG, Human Protein Atlas, and Metabolic Atlas.^19^, ^20^, ^21^

### 2.7 Resin electron microscopy (EPON)

HC ICOs were fixed in half strength Karnovsky fixative (2.5% Glutaraldehyde (EMS) + 2% Formaldehyde (Sigma)) pH 7.4 at RT for 2 hours. ICOs were rinsed and stored in 1 M phoshate buffer pH 7.4 at 4°C until further processing. Postfixation was performed with 1% OsO_4_, 1.5% K_3_Fe(III)(CN)_6_ in 1 M phoshate buffer pH 7.4 for 2 hours. ICOs were then dehydrated in a series of aceton, and embedded in Epon (SERVA). Ultrathin sections were cut (Leica Ultracut UCT), collected on formvar and carbon coated transmission electron microscopy (TEM) grids, and stained with uranyl acetate and lead citrate (Leica AC20). Micrographs were collected on a JEM1010 (JEOL) equipped with a Veleta 2 k × 2 k CCD camera (EMSIS, Munster, Germany) or on a Tecnai12 (FEI Thermo Fisher) equipped with a Veleta 2 k × 2 k CCD camera (EMSIS, Munster, Germany) and operating SerialEM software.

## 3 RESULTS

### 3.1 Organoid generation and culture

**FIGURE 1:**
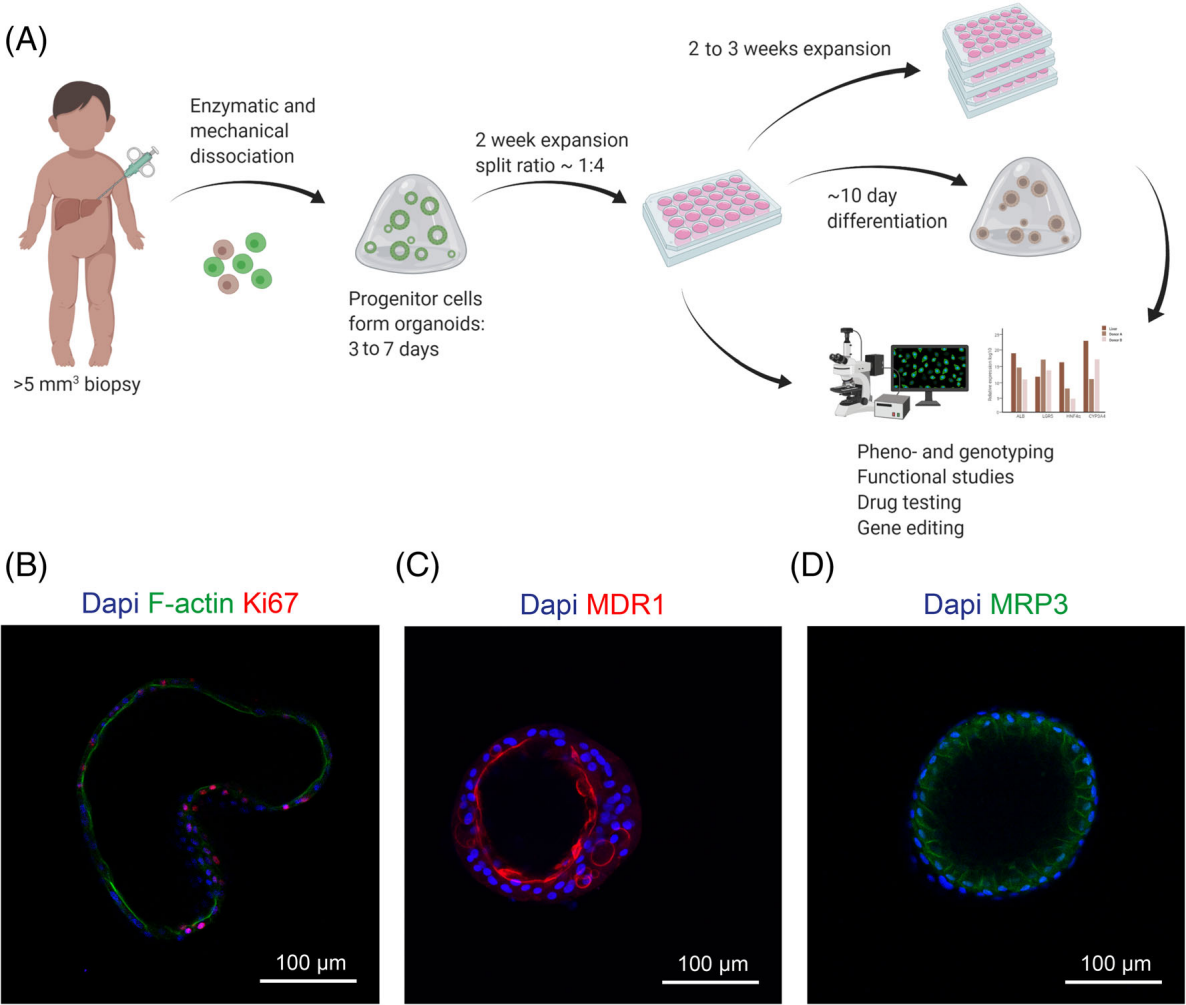
Overview of organoid generation, use and characteristics. (A) Organoid generation from biopsy to *in vitro* culture can be achieved within 3 weeks, whereafter pheno‐ and genotyping, functional assays, drug testing, gene editing, and/or differentiation can take place. (B) Organoids express proliferation marker Ki67 and apical marker F‐actin in expansion conditions (EM). After differentiation (DM), organoids condense and display more mature liver functions and stronger polarization, as exemplified by the apical transporter MDR1 and the basolateral transporter MRP3. Nuclear staining is shown with Dapi

ICO generation is simple and can be achieved within 3 to 7 days (Figure 1A) from a biopsy of approximately 5 mm^3^. This small amount of tissue is often available from clinical procedures, without additional surgical intervention. Tissue can be dissociated and seeded for culture directly, or be stored at 4°C up to 1 week or at −80°C in cell freezing medium for long‐term storage prior to ICO generation (personal experience). Thereby exchange of tissue between different hospitals and research centers is facilitated. Tissue digests are cultured in Matrigel droplets, wherein progenitor cells self‐organize into polarized ICOs within 3 to 7 days (Figure 1). Apical markers such as MDR1 localize to the inner membrane of the cystic ICO, while basolateral markers such as MRP3 localize to the basolateral domain. On average, ICO formation efficiency is 80% to 90% for both healthy and patient donors (Figure S1A). The majority reaches 90% confluency 7 days post isolation, with a variance of up to 2 weeks since a minority of donor ICOs performs poorly (Figure S1A). Once confluent, ICOs are removed from Matrigel and passaged at an average split ratio of 1:5, resulting in a full well plate 3 weeks post isolation (Figures 1A and S1B). This quantity suffices for initial gene expression, histology and functional analyses, while remaining ICOs can be expanded further for biobanking, functional studies, genetic engineering, and/or differentiation (Figure 1A).^6^, ^22^ Throughout differentiation ICOs condense, displaying a thicker cell layer than in EM (Figure 1B), and hepatic characteristics such as albumin production, urea elimination, and phase I enzyme activity increase.^6^

### 3.2 Metabolic processes and disease to study in patient ICOs

ICOs have been shown to retain aspects of liver function *in vitro* such as glycogen storage and albumin secretion.^6^ We further investigated which general and liver metabolic functions can be studied with ICOs.

### 3.3 Basic metabolism

Basic metabolic processes such as amino acid and energy metabolism occur in various tissues throughout the body. One example is branched chain amino acid (BCAA) metabolism which is affected in patients suffering from organic acidemia, such as methylmalonic acidemia (MMA). Monogenic defects in the genes methylmalonyl‐CoA mutase (*MMUT*, 609058), metabolism of cobalamin associated A (*MMAA*, 607481), B (*MMAB*, 607568), D (*MMADHC*, 611935), and methylmalonyl‐CoA epimerase (*MCEE*, 608419) lead to disruption of the enzymatic chain that constitutes BCAA metabolism. Consequently, methylmalonic acid, propionic acid, and respective carnitines accumulate in patient organs causing metabolic crisis, neurological symptoms, kidney failure, and blindness.

**FIGURE 2:**
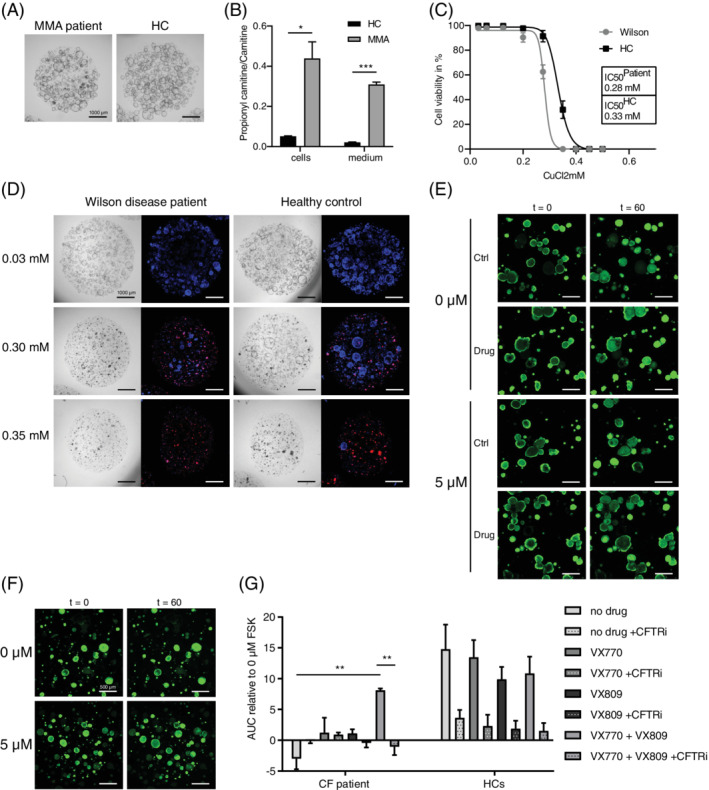
Functional assays revealing disease phenotypes in patient ICOs. (A) Representative culture of MMA patient and HC ICOs under EM. (B) Branched‐chain amino acid metabolism defect exemplified by significantly increased propionylcarnitine to carnitine ratio in ICOs of an MMA patient (n = 2; *, *P*‐value = .0221 in cells; ***, *P*‐value = .0009 in medium). (C) Copper metabolism defect exemplified by increased sensitivity to copper (CuCl_2_) toxicity in ICOs of a Wilson disease patient compared to a HC after 72 hours of exposure (n = 12). (D) Brightfield and IF images of the viability assay in C. showing key concentrations of CuCl_2_. Red signal corresponds to necrosis marker propidium iodide; blue signal indicates DNA. (E‐G) Impaired apical transport can be studied in ICOs as exemplified by defect and rescued CFTR function (***, *P*‐value <.0001) in ICOs of a Cystic fibrosis patient. (E,F) Representative images of CF patient (E) and HC (F) ICO swelling. (G) AUC of CF patient (n = 3) and HC (n = 6) ICO swelling with or without the drugs VX809 and VX‐770 (**, *P*‐value <.0031) and with or without CFTR inhibitor (**, *P*‐value <.0028) at 5 μM FSK relative to 0 μM FSK. AUC, area under the curve; CF, cystic fibrosis; EM, expansion condition; FSK, forskolin; HC, healthy control

As a proof of principle that basic metabolic processes can be studied in ICOs, we established ICOs from an MMA patient with homozygous mut^0^ mutations (c.1280G>T, p.Gly427Val). MMA patient and healthy donor ICOs were similar in growth rates and culture longevity (Figure 2A). LC‐MS/MS acylcarnitine analysis in expanded ICOs cells and culture media revealed significantly increased propionylcarnitine concentrations in patient ICOs and media compared to controls (Figures 2B and S1C,D). However, methylmalonylcarnitine was not detectable in patient and control ICOs (Figure S1C,D), which may reflect insufficient sensitivity of our assay because methylmalonylcarnitine concentrations in plasma from MMA patients can be 50‐fold lower than propionylcarnitine concentrations (personal experience). Propionylcarnitine is an intermediate product in the BCAA metabolism upstream from methylmalonyl‐coenzyme A mutase and is used as a clinical biomarker.^23^, ^24^ Our results suggest that ICOs could serve to study basic metabolic functions such as BCAA metabolism using routine LC‐MS/MS analyses.

### 3.4 Liver specific metabolism

Other metabolic processes, such as metal‐ and drug‐metabolism, are liver specific. Wilson disease (277900) represents a genetic defect in copper metabolism. Mutations in the gene ATPase copper transporting beta (*ATP7B*, 606882) lead to accumulation of toxic amounts of copper in the liver. Patients currently rely on life‐long symptomatic treatment. Therapy development would benefit from organ and patient specific models, that can model the more than 500 different known mutations in *ATP7B*.^25^

Therefore, we investigated copper metabolism in patient ICOs (c.[1288dup(;)1288dup] p.[(Ser430Lysfs*5)(;)(Ser430Lysfs*5)]). Under normal culture conditions no morphological, growth rate, nor longevity differences were observed between healthy control (HC) and Wilson disease patient ICOs (Figure 2D, 0.03 mM CuCl_2_). However, patient ICOs showed increased sensitivity to copper treatment, with an IC_50_ of 0.28 mM CuCl_2_ compared to 0.33 mM in HC ICOs (Figure 2C,D). Putatively, similar storage diseases may be phenotyped with ICOs through provision of relevant substrates.

### 3.5 Functions dependent on 3D structure

Substrate and waste product transport across cells is crucial for many physiologic processes, including bile and mucus metabolism. This process is affected in cystic fibrosis (CF, 219700), a disease caused by mutations in the gene encoding for the CFTR.^26^ This transmembrane protein transports chloride ions to aid in mucus and bile homeostasis. Mutations of the *CFTR* gene (602421) are highly heterogeneous across the population and so are responses to different treatment strategies. Organoids are highly suitable for studying effects of individual *CFTR* mutations due to their patient‐specific origin, unique 3D constellation, and polarity. Substrate and waste product transport across the cell can easily be studied in organoids due to the separated inner apical and outer basolateral domain (Figure 1B‐D). Intestinal organoids are already used to predict medication response for patients using the FIS assay.^16^ Bile excretion from hepatocytes into bile canaliculi reflects a similar process, which is affected in some CF patients and many patients with intrahepatic cholestasis.

We investigated apical transport and medication response in ICOs from a CF patient compound heterozygous for F508del (c.1521_1523delCTT, p.Phe508del) and R1162X (c.3484C>T, p.Arg1162X)(Figure 2E‐G). CF patient ICOs showed impaired swelling in response to forskolin exposure, which corresponds to increased bile viscosity as observed in CF patients. This phenotype was rescued by addition of the corrector and potentiator combination VX809‐770. Using similar approaches, other apical or basolateral functions may also be tested in ICOs.

### 3.6 Metabolic functions not detected in ICO cultures

For some cellular and metabolic functions ICOs appear less suitable. We experienced this for cytosolic aminoacyl‐tRNA synthetase (ARS) deficiencies, an increasingly recognized group of diseases with varying clinical phenotypes. To investigate disease mechanism and improve treatment strategies we established ICOs from a patient with isoleucyl‐tRNA synthetase (*IARS*, 600709, c.1305G>C (p.Trp435Cys), c.3377dup (p.Asn1126fs)) deficiency. The most prominent clinical phenotype of patients, namely dysmaturity and severe failure to thrive,^27^ was closely recapitulated by these ICOs. Concurrently, not enough ICOs could be generated to perform functional assays (Figure S1B,E,F). Thus, faithful disease modeling hampered use of IARS deficient ICOs for unraveling the disease mechanism. Conversely, patient fibroblast growth was sufficient to devise a treatment strategy, which was successfully translated to the clinics.^28^ This indicates that, for some IEMs, alternative patient‐derived cell models should be explored if patient ICOs do not meet the practical requirements, such as expression of genes of interest and cell growth, to unravel disease mechanisms.

Moreover, we anticipate that hyperoxaluria type 1 (PH1, 259900), caused by mutations in the gene alanine‐glyoxylate aminotransferase (*AGXT*, 604285), cannot be studied in ICOs, since *AGXT* is not expressed in healthy ICOs under current culturing conditions (Figure S2D).

To gain broader insight in the metabolic functions that can be studied in ICOs, we investigated the geno‐ and phenotype of ICOs in more detail.

### 3.7 Expression of genes involved in IEMs

Bulk RNA sequencing (RNAseq) was analyzed for IEM expression using the Radboudumc Exome panel for metabolic disorders.^29^ Healthy ICO data were compared to whole liver and fibroblasts, the latter of which is the most commonly used in vitro model for IEMs.^1^, ^30^, ^31^, ^32^, ^33^

**FIGURE 3:**
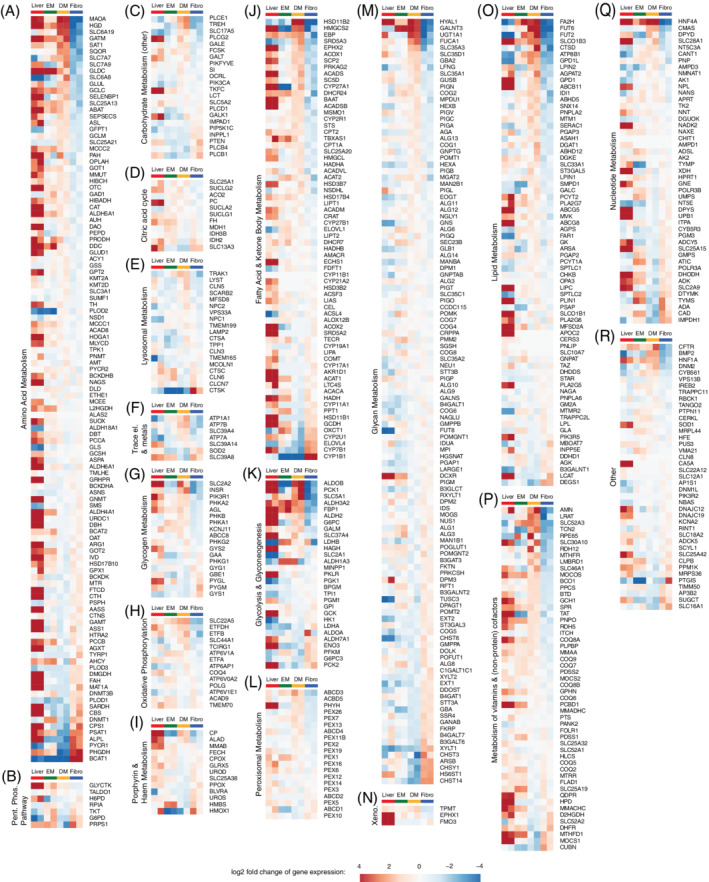
Visualizations of log2 fold changes in gene expression of IEM genes in expanded (EM, n = 2) and differentiated (DM, n = 2) ICOs, fibroblasts (Fibro, n = 2) and whole liver tissue (n = 2). log2 fold changes are relative to the mean expression of the genes across all fibroblast and ICO DM samples. IEM genes are divided into metabolic categories Amino Acid Metabolism (A), Pentose Phosphate Pathway (B), Carbohydrate Metabolism (other) (C), Citric acid cycle (D), Lysosomal Metabolism (E), Trace element and Metal Metabolism (F), Glycogen Metabolism (G), Oxidative Phosphorylation (H), Porphyrin and Haem Metabolism (I), Fatty Acid and Ketone Body Metabolism (J), Glycolysis and Glyconeogenesis (K), Peroxisomal Metabolism (L), Glycan Metabolism (M), Xenobiotics Metabolism (N), Lipid Metabolism (O), Metabolism of vitamins and (nonprotein) cofactors (P), Nucleotide Metabolism (Q), Other (R)

We found a great variety in ICO IEM gene expression, confirming our previous notion that ICOs are a suitable *in vitro* model for a specific selection of metabolic categories (Figures 3 and S2A‐B). Interestingly, this variation was also observed within each metabolic category of ICOs and fibroblasts. Neither model expressed more than 60% of IEM genes of any metabolic category well (Figure S2B). Good IEM gene expression in ICOs was frequently paired with poor expression in fibroblasts and vice versa (Figures 3 and S2B). This insight could facilitate decision making for future studies.

Nonetheless, expression of 30% of all IEM genes was higher in ICOs than fibroblasts (Figure S2A). These genes constitute a variety of metabolic categories, including oxidative phosphorylation, metal, and amino acid metabolism (Figures 3 and S2B). For example, expression of *MMUT*, *CFTR*, and *ATP7B* in ICOs was comparable to that in the liver, whereas fibroblasts displayed lower expression (Figure 3A,F,R). This suggests that ICOs are suitable to study diseases related to these genes, as confirmed by our functional assays (Figure 2).

In fibroblasts, 22% of all IEM genes were better expressed than in ICOs (Figure S2A). Examples include *CPS1*, *CBS*, and *CYP7B1* (Figure 3A,J). Poor expression of *CPS1* and *CBS* in ICOs highlights that ICOs fail to represent certain liver‐specific genes despite being derived from this organ. This led us to investigate the cell characteristics of ICOs using electron microscopy.

### 3.8 CO cell morphology and organelles

**FIGURE 4:**
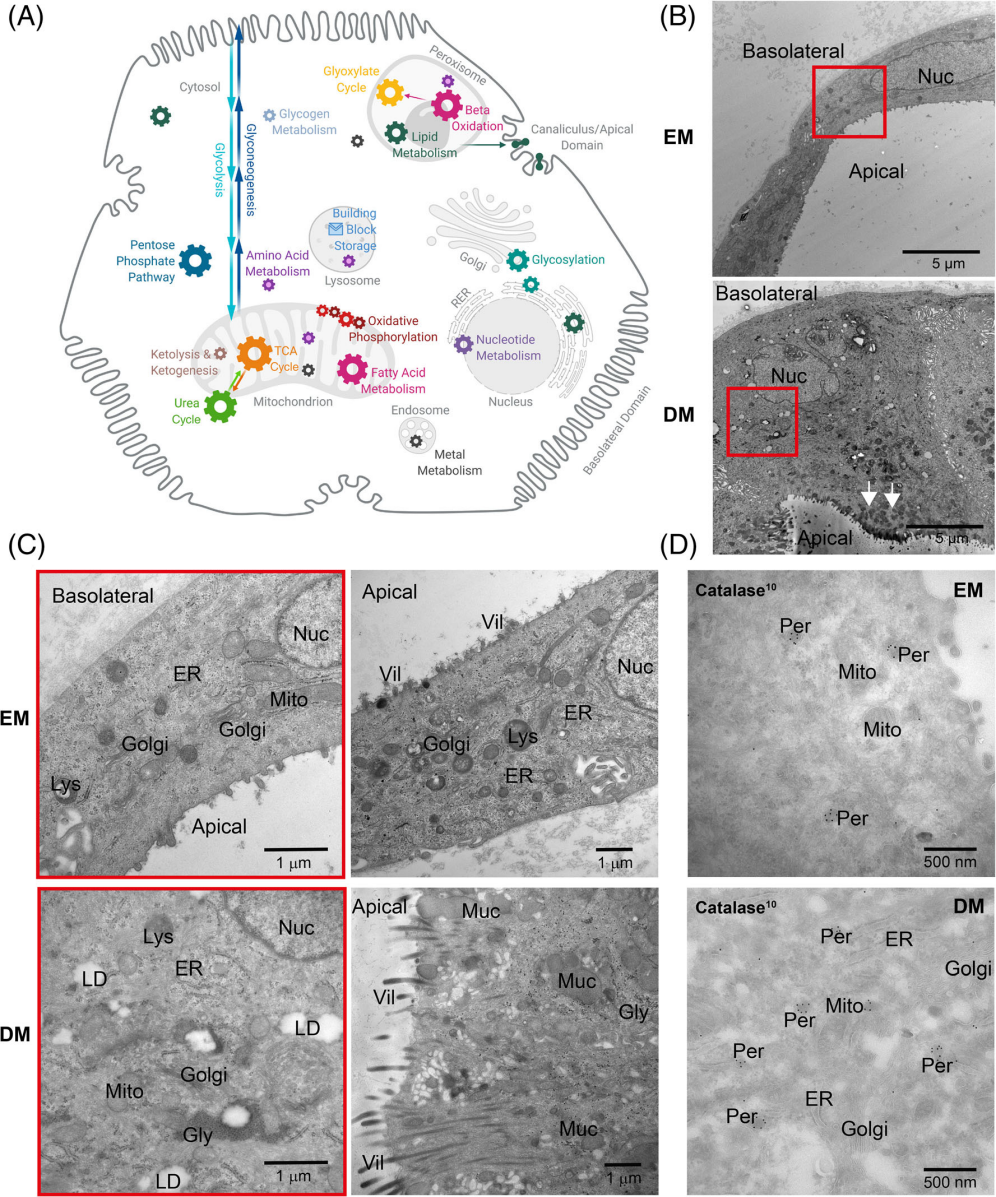
(A) Schematic representation of a hepatocyte showing metabolic functions and approximate organelle locations. Organelle sizes not to scale. (B) TEM images of HC ICO cells in EM and DM displaying differences in ICO wall thickness. Arrows indicate mucus fields. Red squares indicate magnification regions shown in C. (C) TEM images of HC ICO cells in EM and DM displaying the major organelles visible in these cells (left), mucus and villus presence (right), and peroxisomes (D). TEM, transmission electron microscopy; DM, differentiation condition; EM, expansion condition; Gol, golgi; Gly, glycogen rosettes; Lys, lysosomes; Mit, mitochondria; Nuc, nucleus; Per, peroxisomes; ER, endoplasmic reticulum; Vil, villi

Several metabolic functions depend on specific cell organelles (Figure 4A). Hence, ICOs should exhibit all organelles necessary for the function to be studied.

TEM analysis showed that healthy ICO cells in EM and DM have a nucleus, mitochondria, golgi, lysosomes, rough endoplasmic reticulum, and villi (Figure 4B,C). Catalase‐stained peroxisomes were identified in both conditions (Figure 4D). However, catalase signals in DM were mostly found in golgi‐budding vesicles, which could not be found in EM or control HepG2 cells, thus suggesting immature peroxisomes in DM. Peroxisomes in EM and DM ICOs were significantly smaller than in HepG2 cells, a cell model widely viewed to have high similarity to primary human hepatocytes (Figure S3A,B).

Notably, EM ICO cells displayed some liver functionality as they contained glycogen rosettes. Moreover, desmosomes and interdigitations between cells and more compact villi facing ICO lumen indicate apical domains needed for bile salt secretion (Figure S3C). In contrast, the basolateral domain appeared smooth and without villi, concurring with immunofluorescence analyses (Figure 1B).

Interestingly, differentiation induced formation of large mucus fields, located toward the apical domain (Figure 4B). Apical villi in DM ICOs appeared slightly longer and were organized in bundles, putatively to make way for mucus secretion, as would be rather compatible with biliary than hepatic differentiation (Figure 4C, right). Moreover, DM cells showed more lipid droplets compared to EM (Figures 4C and S3C). Occasionally, we found DM cells with less mucus and shorter, more compact villi. Together with the variable transcriptome profile, ICOs appear to undergo heterogenic maturation with current DM, showing cholangiocyte and hepatic characteristics.

## 4 DISCUSSION

Mechanistic studies and treatment development for rare diseases are limited by the small number and geographic distribution of patients. Recently developed organoid models promise exciting possibilities for patient‐specific preclinical studies. However, it is currently unknown for which specific metabolic functions and diseases ICOs can be effectively used. To address this, we present our experience with ICOs and evaluate the potential and limitations of patient‐derived ICOs to study metabolic functions.

We show that ICOs can be used to study basic metabolism, more specific hepatic functions, and transport functions, exemplified respectively by pathways of BCAA metabolism, copper metabolism, and chloride transport. We noticed that IEM gene expression in ICOs and fibroblasts varied within each metabolic category. Cell model choice should be done case by case with focus on the IEM gene/pathway of interest. ICO transcriptome variance was supported by TEM analysis which revealed that ICOs are composed of intermediate cell types with progenitor cell, hepatic, and biliary characteristics. Likely, the cells' ductal origin as well as the environmental stimuli offered to ICOs promote this intermediary cell type. Indeed, it is well known that environmental stimuli affect cell fate.^34^, ^35^, ^36^, ^37^, ^38^ Adjustments in DM and hydrogel composition could favor expression of some as of yet absent metabolic functions. We and other research groups are currently exploring different approaches to achieve improved separate hepatic or cholangiocyte differentiation of ICOs.^35^ We anticipate new insights to arise and be adopted widely in the coming decade. Until then, this paper addresses the recurring queries on ICO use for current clinical and research questions.

Several cell models are available for IEM research, including, ICOs, fibroblasts, cell lines, primary hepatocytes, and induced pluripotent stem cells (Table 1). Most of these cell models are suitable for personalized medicine approaches and biobanking. Cell model choice will depend on the specific study goal, availability of patient cells through skin and/or liver biopsy, representation of metabolic function, costs, and expertise. Although ICOs require some expertise and investment, they score high for other categories. ICOs not only express a variety of IEM genes, but time to first assay and ease of handling are additional advantages. Moreover, ICO culture is versatile; both long‐term 3D and 2D transwell cultures are possible, as previously reported for gut organoids.^39^

**TABLE 1:** Comparison of various cell models for IEM *in vitro* modeling

	ICOs	Fibroblasts	Liver cell lines	PHH	iPSCs
2D culture	+	+	+	+	+
3D culture	+	−	−	−	+/−
Personalized medicine	+	+	−	+/−	+
Long‐term cultures	+	+	+	−	+
Biobanking potential	+	+	+	+/−	+
Ease of handling/assays	+	+	+	−	−
Time to first assays	3 weeks	6 weeks	Immediate	<1 week	6 weeks
Culturing costs/expertise	High	Low	Low	Intermediate	High
Hepatocyte functions	+/−	−	+	++	+/−
Cholangiocyte functions	+/−	−	−	−	+/−
IEM functions	+/−	+/− −	++/−	++/−	+/−

Abbreviations: iPSCs, induced pluripotent stem cells; PHH, primary human hepatocytes; +, applicable; ++, very applicable; −, not applicable; (+)+/−(−), applicable with limitations, while ++ and − − indicate better or worse; 2D, two‐dimensional; 3D, three‐dimensional.

Moreover, ICO generation and differentiation does not require genetic reprogramming or immortalization.^40^, ^41^ Previous *in vitro* copper metabolism studies were all performed in genetically induced fibroblasts or embryonic stem cells.^42^, ^43^, ^44^ We show this can also be done in ICOs which retain the original patient (epi)genome.^6^, ^41^, ^45^ Further studies are needed to determine applicability of Wilson disease ICOs for personalized drug testing.^46^, ^47^

To study basic metabolism, a hepatic phenotype is not required. Although transcriptome analysis favors ICOs to study MMA (Figures 3 and S2B,C), several studies have reported successful phenotyping of MMA in patient fibroblasts and immortalized kidney tubule cells, derived from patient urine.^48^, ^49^, ^50^ This illustrates that lower expression of genes does not necessarily result in absence of a disease phenotype. In ICOs derived from MMA patients, we discerned significantly increased concentrations of the clinical biomarker propionylcarnitine, which represents a first step toward studying MMA treatment response in a personalized setting in ICOs.

Importantly, ICO differentiation capacity varies between donors. It is currently unclear whether this relates to a specific biopsy, isolation or a donor's genetic background or age. It has been shown that extrahepatic cholangiocyte progenitors cannot differentiate to hepatocytes, indicating that the location of cholangiocyte progenitors is crucial for their differentiation potential.^51^, ^52^ Yet, this interdonor variability does not hamper studying intradonor differences after treatment.

When patient material is scarce, patient mutations may be introduced in cells.^42^ Current CRISPR‐based technologies are also applicable to ICOs.^22^, ^53^, ^54^ Moreover, mechanistic insight can be achieved by editing different genes in a pathway. Evidently, artificial IEM ICO models may be helpful in investigating the gene in isolation, but not for personalized strategies.

Complete absence of a key pathway gene is likely to hamper studies thereof. For example, we expect oxalate metabolism studies in ICOs to be impeded by absence of expression of the peroxisomal *AGXT* gene. Peroxisomal assembly genes such as the *PEX*, *PPAR*, and *ABCD* families were well expressed, suggesting availability of peroxisome machinery in ICOs. In contrast, electron microscopy analysis revealed a reduced peroxisome size. Peroxisome biogenesis is highly plastic and dependent on nutrient availability and culture confluency.^55^, ^56^, ^57^, ^58^, ^59^ This has been shown for HepG2 peroxisomes which transiently become tubular rather than spherical during periods of rapid growth.^59^ The smaller peroxisomes in ICOs might represent this transient morphology in peroxisome biogenesis. Provision of relevant substrates in culture media as well as improved differentiation conditions might promote peroxisome maturation and expression of yet absent genes.

Initially ICOs were described as a liver model to study specific hepatic functions. Concurrently, ICOs were shown to eliminate urea, metabolize drugs, and secrete albumin.^6^, ^14^, ^15^ ICOs are derived from the oval cell, or bi‐potent liver progenitor, but current methods do not suffice to generate a pure population of mature hepatocytes. With current methods, ICOs are suitable for studying a selection of basic, hepatic, and cholangiocyte metabolic functions. Prior to using ICOs for a specific research question, expression of the corresponding pathway and/or function should be considered. If the full spectrum of mature hepatic functions is required, a different cell model is more suitable.

Recently, hepatic liver organoids (HLOs) were established from foetal hepatocytes.^60^ These showed an improved hepatic phenotype compared to ICOs. We anticipate that generation of HLOs from pediatric and adult tissue will provide an improved hepatic patient‐specific *in vitro* model and will fill some gaps in patient‐related IEM research.

To conclude, we provide an overview of metabolic functions and IEMs, which can be studied with ICOs. Presence of mitochondria, lysosomes and the ER combined with good gene expression in energy, amino acid, and lipid metabolism suggest that ICOs are suitable to study related functions and diseases. Furthermore, the 3D nature of ICOs renders the model highly suitable for transepithelial transport studies. We present several functional assays with which to study drug responses preclinically. This is especially relevant for IEMs where global patient numbers and geographic distribution do not allow for standard clinical drug testing. Our transcriptome data may be of help to decide whether ICOs are a suitable model for a specific research question. For diseases that can currently not be studied with ICOs, we anticipate that improved culturing conditions and/or adult HLOs will be available in the near future.

### ACKNOWLEDGENT

The authors are grateful for the collaborative “United for Metabolic Diseases (UMD)” efforts to improve care for patients with (genetic) metabolic diseases. Moreover, the authors thank prof. Dr. Jeffrey Beekman for kindly providing relevant medications and inhibitors for CF related experiments. This work was supported by Metakids funding (to Sabine A. Fuchs), a Clinical Fellows grant (40‐00703‐97‐13537 to Sabine A. Fuchs) and a grant from the research program Applied and Engineering Sciences (15498 to Bart Spee), both from The Netherlands Organization for Health Research and Development.

## CONFLICT OF INTEREST

The authors declare no potential conflict of interest.

## Supporting information


**Appendix** S1: Supporting Information

**Appendix** S2: Supporting Information
