# Supplementary material for: The potential and limitations of intrahepatic cholangiocyte organoids to study inborn errors of metabolism
Source: J Inherit Metab Dis. 2021 Nov 3;45(2):353–65. doi: 10.1002/jimd.12450 (PMC9298016; doi:10.1002/jimd.12450)
Supplement: Supplementary file 2 — Appendix S2: Supporting Information [file JIMD-45-353-s001.docx]

Supplemental Data

# Materials and Methods extended

## Organoid generation and culture

Briefly, liver cells were isolated by mechanical fragmentation, followed by washing with cold AdDMEM/F12 (Gibco) containing 1% (v/v) penicillin-streptomycin (Gibco), 1% (v/v) GlutaMax (Gibco), 10 mM HEPES (Gibco). Subsequently, the tissue was enzymatically digested in Hank’s Balanced Salt Solution (Gibco) containing 0.25 mg/ml collagenase D, 0.1 mg/ml DNase I for 20 min at 37°C, centrifuged at 1500 rpm at 4°C and supernatant removed. Throughout this process the digest was not filtered but plated for culture immediately. Initial culture after isolation was done in seeding medium (SM) containing AdDMEM/F12, 1% (v/v) penicillin-streptomycin, 1% (v/v) GlutaMax, 10 mM HEPES, 2% B27 without vitamin A (Gibco), 1.25 mM N-Acetylcysteine (Sigma), 10 mM nicotinamide (Sigma), 10 nM gastrin (Sigma), 10% RSPO1 (v/v) conditioned media (homemade), 50 ng/ml EGF (Peprotech), 100 ng/ml FGF10 (Peprotech), 25 ng/ml HGF (Peprotech), 5 mM A83-01 (Tocris), 10 mM forskolin (Tocris), 25 ng/ml Noggin (Peprotech), 30% (v/v) Wnt conditioned media (CM, homemade), 10 μM Y27632 (Sigma Aldrich) and 2 μM human embryonic stem cell cloning recovery solution (hES, Stemgent). Differentiation media (DM) contained AdDMEM/F12 medium supplemented with, 1% B27, EGF (50 ng/ml), 1.25 mM N-Acetylcysteine, gastrin (10 nM), HGF (25 ng/ml), FGF19 (100 ng/ml, Peprotech), A8301 (500 nM), DAPT (10 μM, Selleckchem), BMP7 (25 ng/ml), and dexamethasone (30 μM, Sigma-Aldrich).

## Immunohistochemistry

Prior to harvesting ICOs, cultures were incubated with Cell Recovery Solution (Corning) at 4°C for 30 min. Thereafter ICOs were collected, briefly washed and the pellet was resuspended in 4% buffered formaldehyde (Klinipath) and incubated at room temperature (RT) for 45 min. Fixated ICOs were stored in 70% ethanol at 4°C until further use.

## RNA sequencing and analysis

Total RNA was isolated from two HC ICO cultures using Trizol LS reagent (Invitrogen) and stored at –80°C until further processing. ICO mRNA was isolated using Poly(A) Beads (NEXTflex). Sequencing libraries were prepared using the Rapid Directional RNA-Seq Kit (NEXTflex) and sequenced on a NextSeq500 (Illumina) to produce 75 base long reads (Utrecht DNA Sequencing Facility). Read mapping of all data sets was performed on the Galaxy web platform (usegalaxy.eu)^1^ using Gencode human reference genome sequence release 33 (GRCh38.p13), Gencode comprehensive gene annotation v33, and RNA STAR tool (Galaxy Version 2.7.2b). Read counts were obtained using “--quantMode GeneCounts” option in STAR. Count normalization and variance-stabilizing transformation were performed using DESeq2 package (v. 1.30.1), followed by vst transformation and quantile normalization. PCA and pearson correlation analysis showed no observable study-specific batch effects (data not shown).

## Immuno-Electron microscopy

HepG2 cells (ATCC, clone HB-8065) were grown on 100 mm petri dishes in MEM Hanks supplemented with 10% fetal bovine serum (Sigma), 2 mM L-glutamine (Invitrogen) and 100 units per 100 μg ml−1 penicillin/streptomycin (Invitrogen) at 37 °C/5% CO2. Cells were fixed by adding freshly prepared 4% w/v PFA (Polysciences) in 0.1 M phosphate buffer (pH 7.4) to an equal volume of culture medium for 5 min, followed by post-fixation in 4% w/v PFA at 4 °C overnight. Ultrathin cryosectioning and immunogold labelling were performed as previously described.^2^ HC ICOs of one donor were fixed by adding freshly-prepared 4% formaldehyde (Sigma-Aldrich) in 0.1 M phosphate buffer (pH 7.4) in the presence or absence of 0.2% glutaraldehyde (Polysciences Inc.) to an equal volume of culture medium. After 10 min fixative was refreshed to continue fixation for 2 h. Cells were stored in 1% formaldehyde at 4°C until further processing. Embedding, ultrathin cryosectioning and immunogold labelling were performed as described.^2^ In brief, fixed ICOs were washed with 0.05 M glycin in PBS, embedded in 12% gelatin in PBS, 37°C, solidified on ice and cut into small blocks. Cryoprotection was performed by infiltrating blocks overnight in 2.3 M sucrose at 4°C. Blocks were mounted on aluminium pins and frozen in liquid nitrogen. Ultrathin cryosections (60 nm) were cut (Leica EM UC7), transferred on TEM grids with a 1:1 mixture of 2.3 M sucrose and 1.8% methylcellulose, and immunolabelled using primary antibodies (Table S3). Primary antibodies were detected by Protein A conjugated to 10 nm or 15 nm gold particles (Cell Microscopy Core, Utrecht, The Netherlands). Pictures were collected on aJEM1010 (JEOL) equipped with a Veleta 2k×2k CCD camera (EMSIS) or on a Tecnai12 (FEI Thermo Fisher) running SerialEM software.^3^ Quantifications of catalase labelled organelles of 2 cryosections per condition were performed in Fiji.^4^

*Table S1. Primary antibodies for immunohistochemistry*

| **Antigen** | **Supplier** | **Cat. number** | **Raised in** | **Dilution** | **Incubation** |
| --- | --- | --- | --- | --- | --- |
| F-actin | Life Technologies | A12379 | - | 1:400 | o/n 4°C |
| Ki67 | Abcam | ab16667 | rabbit | 1:100 | o/n 4°C |
| MDR1 | Novus bio | NBP1-90291 | rabbit | 1:500 | o/n 4°C |
| MRP3 | Abcam | ab3375 | mouse | 1:100 | o/n 4°C |

Table S2. Secondary antibodies for immunohistochemistry

| **Antigen** | **Supplier** | **Cat. number** | **Raised in** | **Dilution** | **Incubation** |
| --- | --- | --- | --- | --- | --- |
| Anti-mouse Alexa 488 | Life technologies | A11029 | goat | 1:100 | o/n 4°C |
| Anti-rabbit Alexa 568 | Life technologies | A11036 | goat | 1:100 | o/n 4°C |

Table S3. Primary antibodies for immuno-electron microscopy

| **Antigen** | **Supplier** | **Cat. number** | **Raised in** | **Dilution** | **Incubation** |
| --- | --- | --- | --- | --- | --- |
| α-Catalase | Rockland | 200-4151 | rabbit | 1:1500 | 1h RT |
| α-Lysozyme | Dako | A0099 | rabbit | 1:1000 | 1h RT |

Table S4. Primer for quantification of AGXT gene expression

| **Primer name** | **sequence (5'-3')** | **Tm** | **use** |
| --- | --- | --- | --- |
| AGXT-F1 | CATCTCCTTCAGTGACAAGG | 59 | qPCR |
| AGXT-R1 | ACTTGATGTCCAGGTAGAAGG | 59 | qPCR |

Figure S1. Healthy and patient ICO behaviour. A. Growth rates of various HC and patient ICOs. B. ICO passage ratios observed during maintenance culture of one IARS, one MMA, one Wilson disease and one CF patient and 12 HCs. C. & D. Acylcarnitines detected during LC-MS/MS analysis of ICO cells (C) and culture media (D) of one MMA patient and one HC (n=2). (Acyl)carnitines were identified as follows: C0, free carnitine; C2, acetylcarnitine; C3, propionylcarnitine; C4, butyrylcarnitine; C3-DC, malonylcarnitine; C4-DC, succinyl/methylmalonylcarnitine. E. & F. Poor growth of ICOs of an IARS patient compared to a HC.


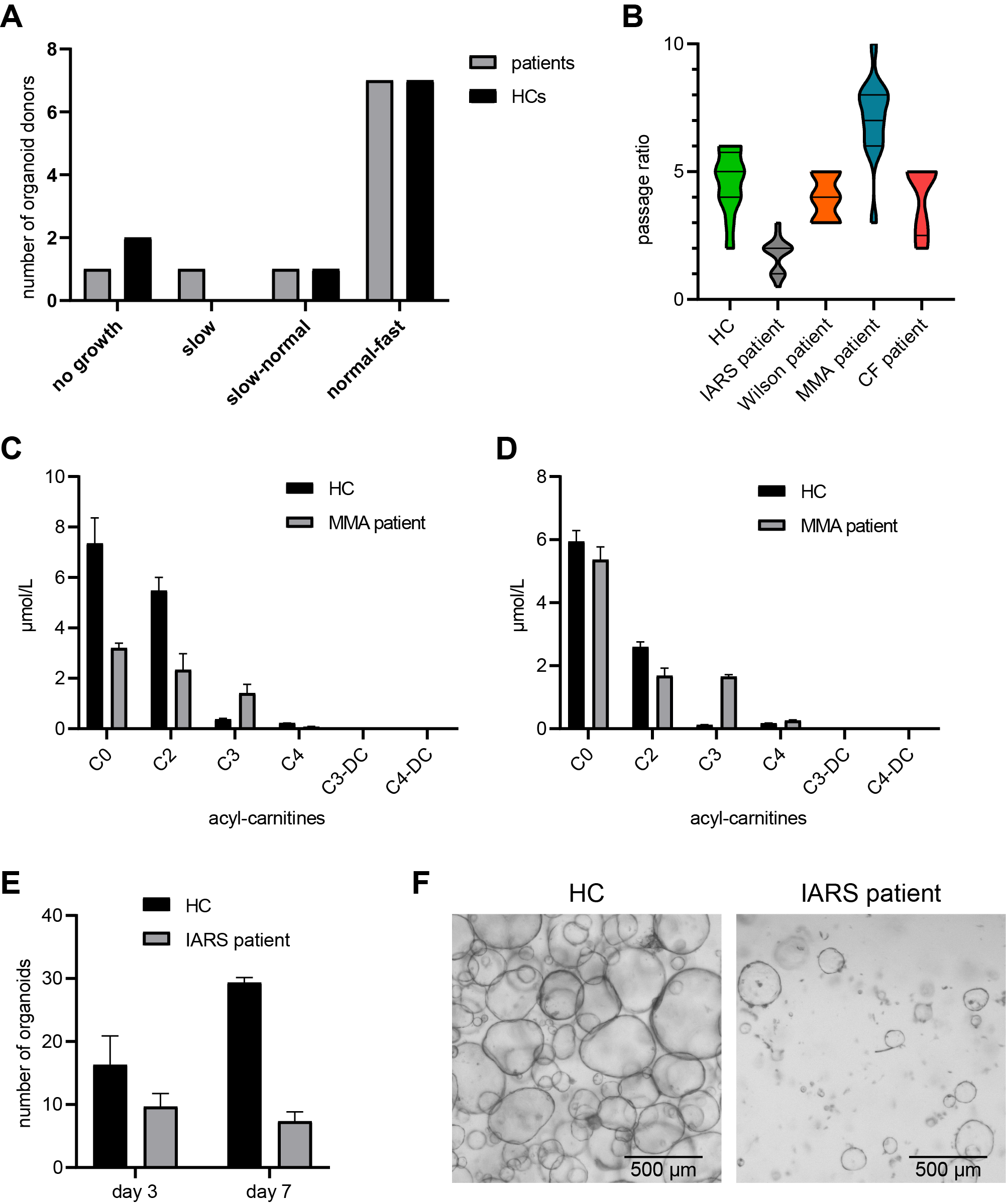


Figure S2. Quantification of the expression of metabolic categories in HC ICOs (n=2) and healthy fibroblasts (n=2). A. Percentages of total IEM genes which are better expressed in HC ICOs, fibroblasts or equally in both cell models. B. Percentages of IEM genes better expressed in HC ICOs (black) and fibroblasts (grey) displayed for each metabolic category. C. Visualizations of log2 fold change expression genes involved in branched chain amino acid metabolism in expanded (EM) and differentiated (DM) HC ICOs, fibroblasts and whole liver. D. Expression of the gene AGXT is absent in HC ICOs.


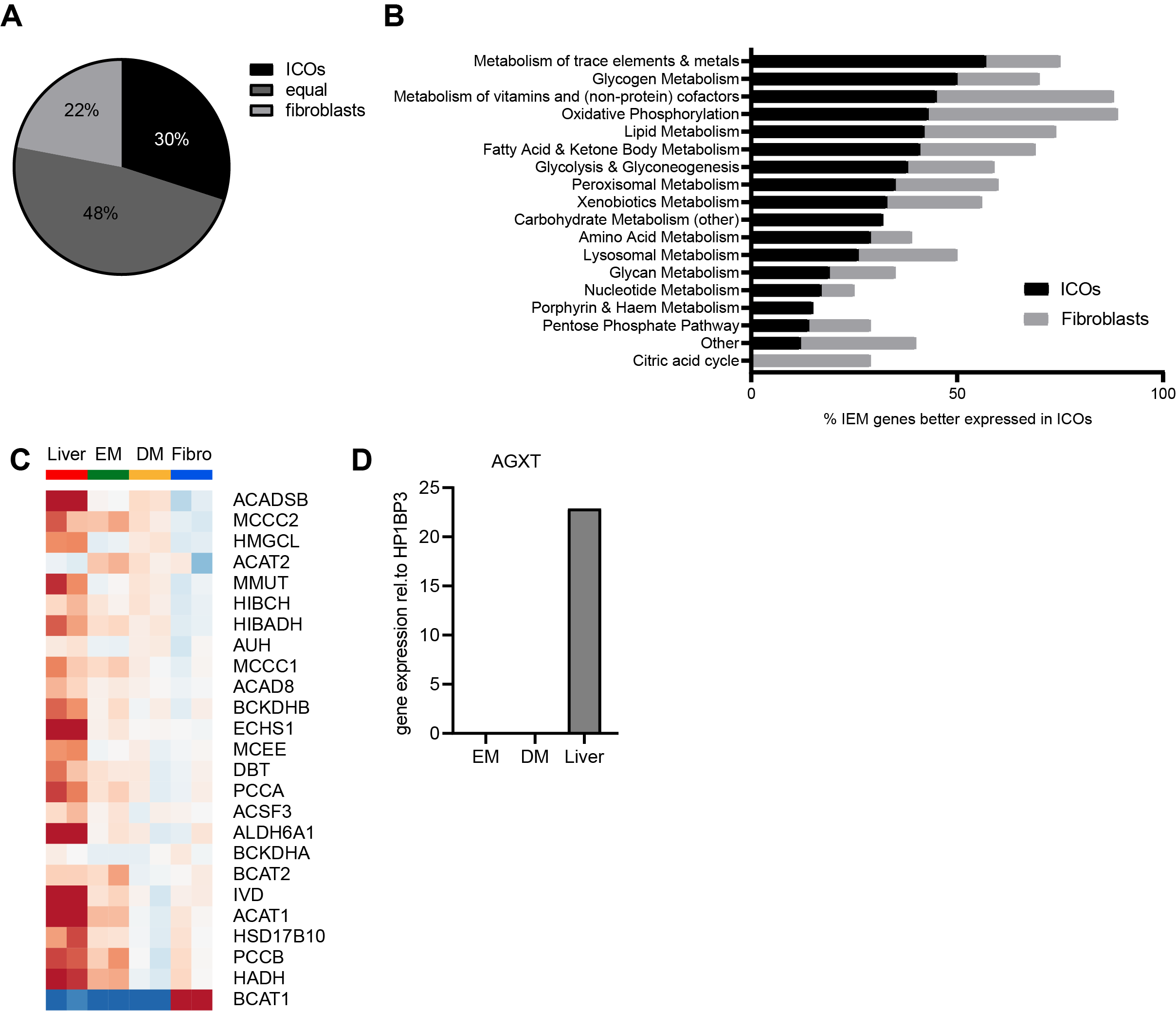


Figure S3. TEM images of ICO and HepG2 cells. A. Peroxisome size in ICOs is significantly smaller than in HepG2 (***, p-value = 0.007; ****, p-value <0.001). B. Catalase-stained peroxisomes in HepG2. C. Macroviews of EM and DM ICO cells reveals desmosomes (arrows) and interdigitations between cells. DM ICOs contain cells resembling hepatocyte like cells (HLC) and mucus producing cells (MPC).


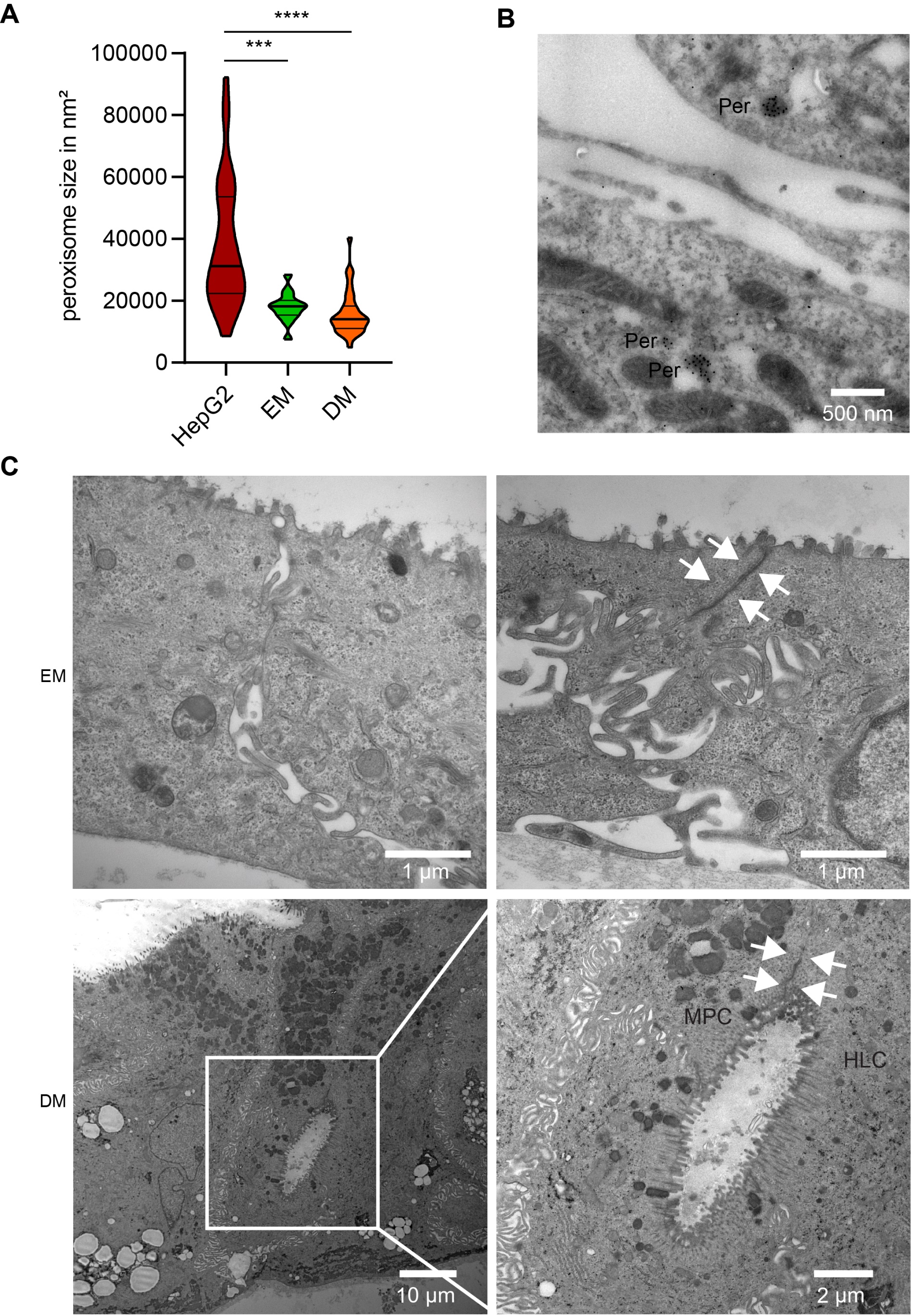


## References

1. Afgan E, Baker D, Batut B, et al. The Galaxy platform for accessible, reproducible and collaborative biomedical analyses: 2018 update. Nucleic Acids Res. 2018;46(W1):W537-W544. doi:10.1093/nar/gky379

2. Slot JW, Geuze HJ. Cryosectioning and immunolabeling. Nat Protoc. 2007;2(10):2480-2491. doi:10.1038/nprot.2007.365

3. Mastronarde DN. Automated electron microscope tomography using robust prediction of specimen movements. Published online 2005. doi:10.1016/j.jsb.2005.07.007

4. Schindelin J, Arganda-Carreras I, Frise E, et al. Fiji: An open-source platform for biological-image analysis. Nat Methods. 2012;9(7):676-682. doi:10.1038/nmeth.2019
